# P-1600. Improving Diagnostic Stewardship for *Clostridioides difficile* Testing

**DOI:** 10.1093/ofid/ofae631.1767

**Published:** 2025-01-29

**Authors:** Hanna Wardell, Mari M Nakamura, Sarah Jones, David A Johnson, Benjamin Ethier, Stacie Haralambous, Nira R Pollock, Stacy A Kahn, Ana M Vaughan-Malloy

**Affiliations:** Boston Children's Hospital, Boston, Massachusetts; Boston Children's Hospital/Harvard Medical School, Boston, Massachusetts; Boston Children's Hospital, Boston, Massachusetts; Boston Childrens Hospital, Norwood, Massachusetts; Boston Children's Hospital, Boston, Massachusetts; Boston Children's Hospital, Boston, Massachusetts; Boston Children's Hospital, Boston, Massachusetts; Boston Children’s Hospital and Harvard Medical School, Boston, Massachusetts; Boston Children's Hospital, Boston, Massachusetts

## Abstract

**Background:**

*Clostridioides difficile* (*C. difficile*) infection (CDI) is the most common healthcare-associated infection in the US. Use of molecular diagnostics may be contributing to increasing incidence due to the high sensitivity of these testing modalities, leading to over-diagnosis in persons with asymptomatic colonization. Colonization with *C. difficile* is common in early infancy. Laxative use may cause diarrhea and confound CDI diagnosis. Misdiagnosis of CDI can lead to over-treatment, increased hospital costs, prolonged hospital stay, misrepresentation of the true incidence of infection, and hospital penalties. Given the high sensitivity of molecular testing methods, diagnostic stewardship is critical to reduce inaccurate clinical diagnosis and inappropriate treatment.

**Figure 1:**
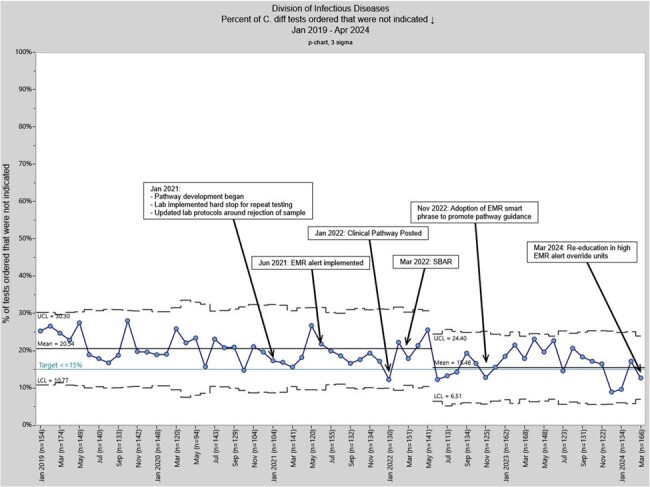
Percent of C. difficile tests ordered that were not indicated. SBAR: Situation, Background, Assessment, and Recommendation; UCL: Upper Control Limit; LCL: Lower Control Limit.

**Methods:**

Starting in January 2021, using Plan-Do-Study-Act (PDSA) cycles, we implemented and evaluated strategies to decrease unindicated C. *difficile* testing at our institution, focusing on patients <1-year-old and those receiving laxatives. Interventions included: (1) Electronic medical record (EMR) alert the time of order entry, (2) rejection of specimens by the laboratory for patients with a negative test within the past 7 days, (3) creation of a clinical pathway for *C. difficile* diagnosis and management, (4) targeted educational sessions to promote appropriate testing and pathway awareness, and (5) feedback to units with high EMR alert override rates.

**Results:**

From January 2021 (baseline) to April 2024, the percentage of *C. difficile* tests sent on patients aged ≤1 year or receiving laxatives decreased from a mean baseline of 20.5% to 15.5% (Figure 1).

**Conclusion:**

This multidisciplinary quality improvement initiative leveraged EMR alerts, clinical practice guidance, laboratory acceptance criteria, and targeted clinician education to reduce unindicated *C. difficile* testing, increasing the likelihood that positive tests reflect disease rather than colonization.

**Disclosures:**

**Ana M. Vaughan-Malloy, MD, MPH**, Asana: Stocks/Bonds (Public Company)|Aurora: Stocks/Bonds (Public Company)|Ayr Wellness: Stocks/Bonds (Public Company)|Bionano Genomics: Stocks/Bonds (Public Company)|Butterfly Network: Stocks/Bonds (Public Company)|Canopy Growth: Stocks/Bonds (Public Company)|Cresco Labs: Stocks/Bonds (Public Company)|CRISPR Therapeutics: Stocks/Bonds (Public Company)|Cronos Group: Stocks/Bonds (Public Company)|Curaleaf Holdings: Stocks/Bonds (Public Company)|Editas Medicine: Stocks/Bonds (Public Company)|Green Thumb Industries: Stocks/Bonds (Public Company)|High Tide: Stocks/Bonds (Public Company)|Iovance Biotherapeutics: Stocks/Bonds (Public Company)|Jushi Holdings: Stocks/Bonds (Public Company)|Moderna: Stocks/Bonds (Public Company)|Organigram Holdings: Stocks/Bonds (Public Company)|Pacific Biosciences of California: Stocks/Bonds (Public Company)|Personalis: Stocks/Bonds (Public Company)|Pfizer: Stocks/Bonds (Public Company)|SNDL: Stocks/Bonds (Public Company)|Terrascend: Stocks/Bonds (Public Company)|Tilray Brands: Stocks/Bonds (Public Company)|Trulieve: Stocks/Bonds (Public Company)

